# Supplementary material for: Frontal Cortex Hyperactivation and Gamma Desynchrony in Fragile X Syndrome: Correlates of Auditory Hypersensitivity
Source: bioRxiv. 2024 Jun 14:2024.06.13.598957. Preprint. [Version 1] doi: 10.1101/2024.06.13.598957 (PMC11195233; doi:10.1101/2024.06.13.598957)
Supplement: Supplement 1 [file NIHPP2024.06.13.598957v1-supplement-1.pdf]

bioRxiv preprint doi: https://doi.org/10.1101/2024.06.13.598957; this version posted June 14, 2024. The copyright holder for this preprint (which was not certified by peer review) is the author/funder, who has granted bioRxiv a license to display the preprint in perpetuity. It is made available under aCC-BY 4.0 International license.

Supporting Information

Supplementary Table 1. Assignment of cortical nodes to region groupings as attributed by the Desikan-Killiany (DK) atlas.

Node	Abbreviation	Cortex	MNI Centroid x	y	z	Vert.
Caudal Anterior Cingulate R	cACC R	RL	4	22	27	68
Isthmus Cingulate L	iCC L	LL	-7	-45	18	81
Isthmus Cingulate R	iCC R	RL	6	-42	19	91
Lateral Orbitofrontal L	LOF L	LPF	-25	30	-19	221
Lateral Orbitofrontal R	LOF R	RPF	23	32	-19	206
Medial Orbitofrontal L	MOF L	LPF	-7	33	-17	155
Medial Orbitofrontal R	MOF R	RPF	4	37	-15	170
Parahippocampal L	paraH L	LT	-26	-29	-21	68
Parahippocampal R	paraH R	RT	24	-30	-19	66
Posterior Cingulate L	PCC L	LL	-5	-15	38	85
Posterior Cingulate R	PCC R	RL	5	-17	39	93
Precuneus L	PCUN L	LP	-9	-59	38	314
Precuneus R	PCUN R	RP	10	-58	38	325
Rostral Anterior Cingulate L	rACC L	LL	-5	39	1	78
Rostral Anterior Cingulate R	rACC R	RL	4	38	3	56
Caudal Middle Frontal L	cMFG L	LF	-37	11	47	224
Caudal Middle Frontal R	cMFG R	RF	37	13	48	186
Insula L	INS L	LT	-38	-2	2	174
Insula R	INS R	RT	36	2	-2	196
Rostral Middle Frontal L	rMFG L	LF	-34	47	17	543
Rostral Middle Frontal R	rMFG R	RF	34	48	17	551
Supramarginal L	SMAR L	LP	-57	-38	34	305
Supramarginal R	SMAR R	RP	54	-31	36	302
Caudal Anterior Cingulate L	cACC L	LL	-5	21	26	48
Inferior Temporal L	ITG L	LT	-53	-36	-22	307
Inferior Temporal R	ITG R	RT	51	-32	-25	316
Middle Temporal L	MTG L	LT	-58	-23	-15	277
Middle Temporal R	MTG R	RT	58	-22	-15	324
Pars Opercularis L	pOPER L	LF	-49	17	14	139
Pars Opercularis R	pOPER R	RF	49	17	14	118
Pars Orbitalis L	pORB L	LPF	-44	39	-14	72
Pars Orbitalis R	pORB R	RPF	43	42	-15	68
Pars Triangularis L	pTRI L	LF	-47	32	1	101
Pars Triangularis R	pTRI R	RF	48	34	2	148

bioRxiv preprint doi: https://doi.org/10.1101/2024.06.13.598957; this version posted June 14, 2024. The copyright holder for this preprint (which was not certified by peer review) is the author/funder, who has granted bioRxiv a license to display the preprint in perpetuity. It is made available under aCC-BY 4.0 International license.

Superior Temporal L	STG L	LT	-55	-12	-4	290
Superior Temporal R	STG R	RT	54	-6	-7	257
Cuneus L	CUN L	LO	-6	-80	19	93
Cuneus R	CUN R	RO	8	-78	20	99
Fusiform L	FUS L	LT	-36	-43	-22	268
Fusiform R	FUS R	RT	35	-41	-23	255
Lateral Occipital L	LOG L	LO	-31	-89	0	371
Lateral Occipital R	LOG R	RO	35	-85	2	367
Lingula L	LING L	LO	-14	-71	-5	246
Lingula R	LING R	RO	13	-67	-4	227
Banks of Sup. Temp. Sulcus L	BSTS L	LT	-53	-45	8	76
Banks of Sup. Temp. Sulcus R	BSTS R	RT	54	-41	10	70
Entorhinal L	ENT L	LT	-26	-5	-33	30
Entorhinal R	ENT R	RT	23	-6	-35	32
Frontal Pole L	FP L	LPF	-7	68	-11	22
Frontal Pole R	FP R	RPF	7	68	-15	30
Inferior Parietal L	IPL L	LP	-42	-71	32	351
Inferior Parietal R	IPL R	RP	46	-63	32	421
Paracentral L	paraC L	LC	-7	-30	57	110
Paracentral R	paraC R	RC	7	-27	57	128
Pericalcarine L	periCAL L	LO	-11	-82	6	109
Pericalcarine R	periCAL R	RO	12	-80	7	110
Postcentral L	postC L	LC	-46	-22	45	333
Postcentral R	postC R	RC	44	-20	46	307
Precentral L	preC L	LC	-41	-9	46	339
Precentral R	preC R	RC	40	-7	46	353
Superior Frontal L	sFG L	LF	-12	30	41	671
Superior Frontal R	sFG R	RF	12	32	41	603
Superior Parietal L	SPL L	LP	-23	-65	50	484
Superior Parietal R	SPL R	RP	24	-65	51	464
Temporal Pole L	TP L	LT	-28	14	-38	38
Temporal Pole R	TP R	RT	27	16	-36	38
Transverse Temporal L	TT L	LT	-46	-23	10	34
Transverse Temporal R	TT R	RT	46	-17	9	23

The MNI coordinates and number of vertices included in each node parcel are also displayed. We considered nodes as replicates within regions within our linear models. Abbreviations: L, Left; R, right; F, frontal; L, cingulate; O, occipital; P, parietal; PF, prefrontal; T, temporal; MNI, Montreal Neurologic Institute; Vert., Number of Vertices.

bioRxiv preprint doi: https://doi.org/10.1101/2024.06.13.598957; this version posted June 14, 2024. The copyright holder for this preprint (which was not certified by peer review) is the author/funder, who has granted bioRxiv a license to display the preprint in perpetuity. It is made available under aCC-BY 4.0 International license.

Supplementary Table 2: Summary of linear mixed model results.

Model	Predictor	numDF	denDF	F-value	p-value
**ITC: 40 Hz**	(Intercept)	1	4,973	16987.5	<.001
	group	1	71	6.9	.011
	sex	1	71	3e-04	.986
	region	13	4,973	38.9	<.001
	group:sex	1	71	3.9	.053
	group:region	13	4,973	1.5	.124
	sex:region	13	4,973	1.7	.058
	group:sex:region	13	4,973	1.9	.025
**ITC: 80 Hz**	(Intercept)	1	4,973	16135.3	<.001
	group	1	71	3.9	.053
	sex	1	71	.8	.367
	region	13	4,973	15.2	<.001
	group:sex	1	71	.6	.456
	group:region	13	4,973	4.6	<.001
	sex:region	13	4,973	1.5	.121
	group:sex:region	13	4,973	1.2	.242
**ITC: Onset**	(Intercept)	1	4,973	7853.2	<.001
	group	1	71	4.3	.042
	sex	1	71	.01	.936
	region	13	4,973	51.9	<.001
	group:sex	1	71	.1	.728
	group:region	13	4,973	2.9	<.001
	sex:region	13	4,973	2.8	<.001
	group:sex:region	13	4,973	1.4	.142
**ITC: Offset**	(Intercept)	1	4,973	14939.9	<.001
	group	1	71	1.5	.218
	sex	1	71	.01	.922
	region	13	4,973	21.8	<.001
	group:sex	1	71	.1	.783
	group:region	13	4,973	1.7	.062
	sex:region	13	4,973	.9	.568
	group:sex:region	13	4,973	1.7	.047
**STP: Gamma1**	(Intercept)	1	4,973	1530204.9	<.001
	group	1	71	12.7	<.001
	sex	1	71	36.4	<.001
	region	13	4,973	434.0	<.001
	group:sex	1	71	4.2	.045
	group:region	13	4,973	2.6	.002
	sex:region	13	4,973	2.6	.001
	group:sex:region	13	4,973	.7	.799
**STP: Gamm2**	(Intercept)	1	4,973	971679.6	<.001

bioRxiv preprint doi: https://doi.org/10.1101/2024.06.13.598957; this version posted June 14, 2024. The copyright holder for this preprint (which was not certified by peer review) is the author/funder, who has granted bioRxiv a license to display the preprint in perpetuity. It is made available under aCC-BY 4.0 International license.

	group	1	71	1.4	.235
	sex	1	71	13.7	<.001
	region	13	4,973	461.9	<.001
	group:sex	1	71	7.8	.007
	group:region	13	4,973	2.5	.002
	sex:region	13	4,973	2.3	.006
	group:sex:region	13	4,973	.5	.943
**STP: Alpha**	(Intercept)	1	4,973	684410.9	<.001
	group	1	71	4.0	.048
	sex	1	71	1.7	.190
	region	13	4,973	171.5	<.001
	group:sex	1	71	6.4	.014
	group:region	13	4,973	2.8	<.001
	sex:region	13	4,973	5.5	<.001
	group:sex:region	13	4,973	9.6	<.001
**ERSP: Gamma1**	(Intercept)	1	4,973	8.2	.004
	group	1	71	2.4	.123
	sex	1	71	3.9	.052
	region	13	4,973	2.1	.011
	group:sex	1	71	.03	.866
	group:region	13	4,973	1.5	.122
	sex:region	13	4,973	.8	.663
	group:sex:region	13	4,973	2.7	<.001
**ERSP: Gamma2**	(Intercept)	1	4,973	4.9	.027
	group	1	71	1.0	.330
	sex	1	71	3.7	.059
	region	13	4,973	2.5	.002
	group:sex	1	71	.2	.659
	group:region	13	4,973	2.7	<.001
	sex:region	13	4,973	2.4	.004
	group:sex:region	13	4,973	2.0	.017
**ERSP: Alpha**	(Intercept)	1	4,973	13.7	<.001
	group	1	71	1.3	.251
	sex	1	71	3.1	.081
	region	13	4,973	6.2	<.001
	group:sex	1	71	6.1	.016
	group:region	13	4,973	1.6	.079
	sex:region	13	4,973	1.0	.403
	group:sex:region	13	4,973	2.8	<.001

A series of linear mixed-effects models (LME) were conducted for each of the ten power and phase response variables (see Methods). To account for individual variation in electrode position,

bioRxiv preprint doi: https://doi.org/10.1101/2024.06.13.598957; this version posted June 14, 2024. The copyright holder for this preprint (which was not certified by peer review) is the author/funder, who has granted bioRxiv a license to display the preprint in perpetuity. It is made available under aCC-BY 4.0 International license.

we opted to perform statistical modeling at the region level and use nodes within a region as replicates. Fixed effects included Group (FXS or TDC), Sex (male or female), and Region (14).

bioRxiv preprint doi: https://doi.org/10.1101/2024.06.13.598957; this version posted June 14, 2024. The copyright holder for this preprint (which was not certified by peer review) is the author/funder, who has granted bioRxiv a license to display the preprint in perpetuity. It is made available under aCC-BY 4.0 International license.

Supplementary Figure 1: Broadband auditory chirp stimulus.

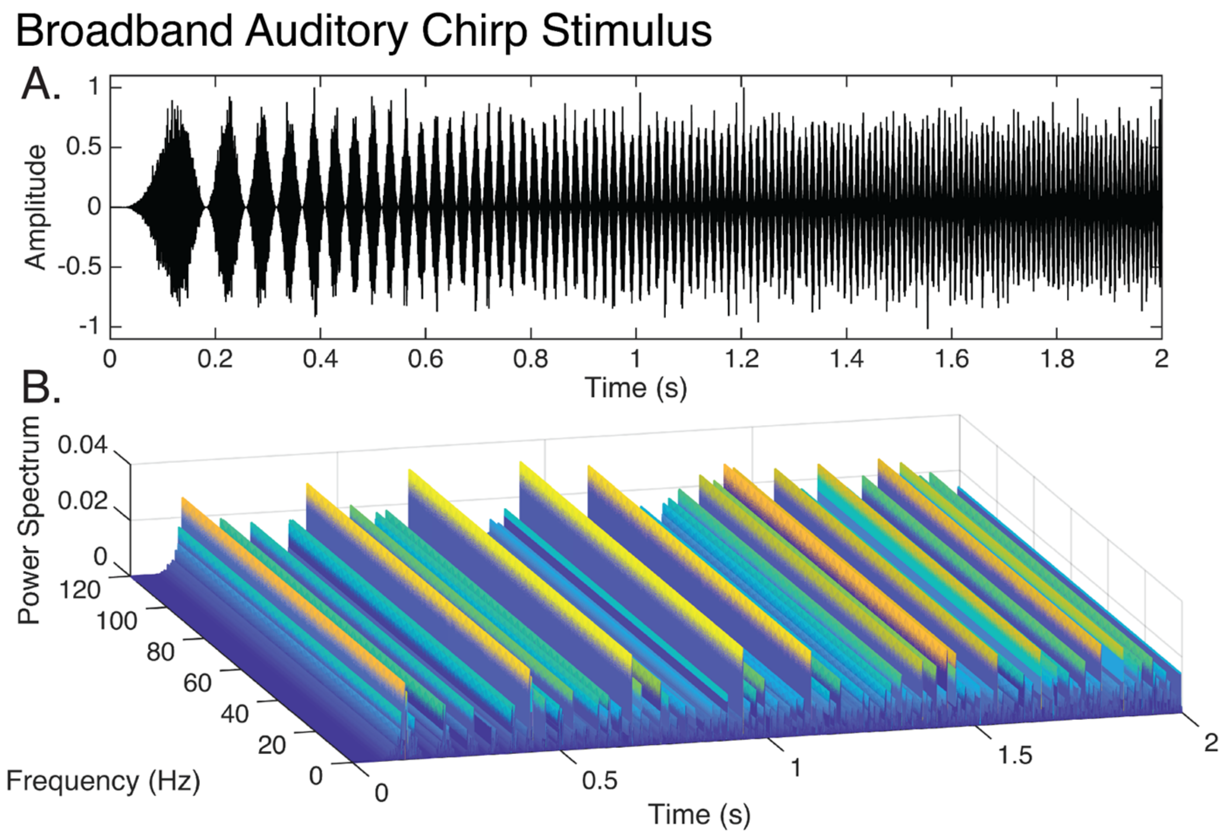

Supplementary Figure 1: A. Plot of amplitude-modulated pink noise carrier waveform. Frequency of modulation increases linearly from 0 to 120 Hz over 2 seconds. B. Waterfall plot depicting broadband power spectrum of broadband chirp stimulus.

bioRxiv preprint doi: https://doi.org/10.1101/2024.06.13.598957; this version posted June 14, 2024. The copyright holder for this preprint (which was not certified by peer review) is the author/funder, who has granted bioRxiv a license to display the preprint in perpetuity. It is made available under aCC-BY 4.0 International license.

Supplementary Figure 2: Atlas Regions

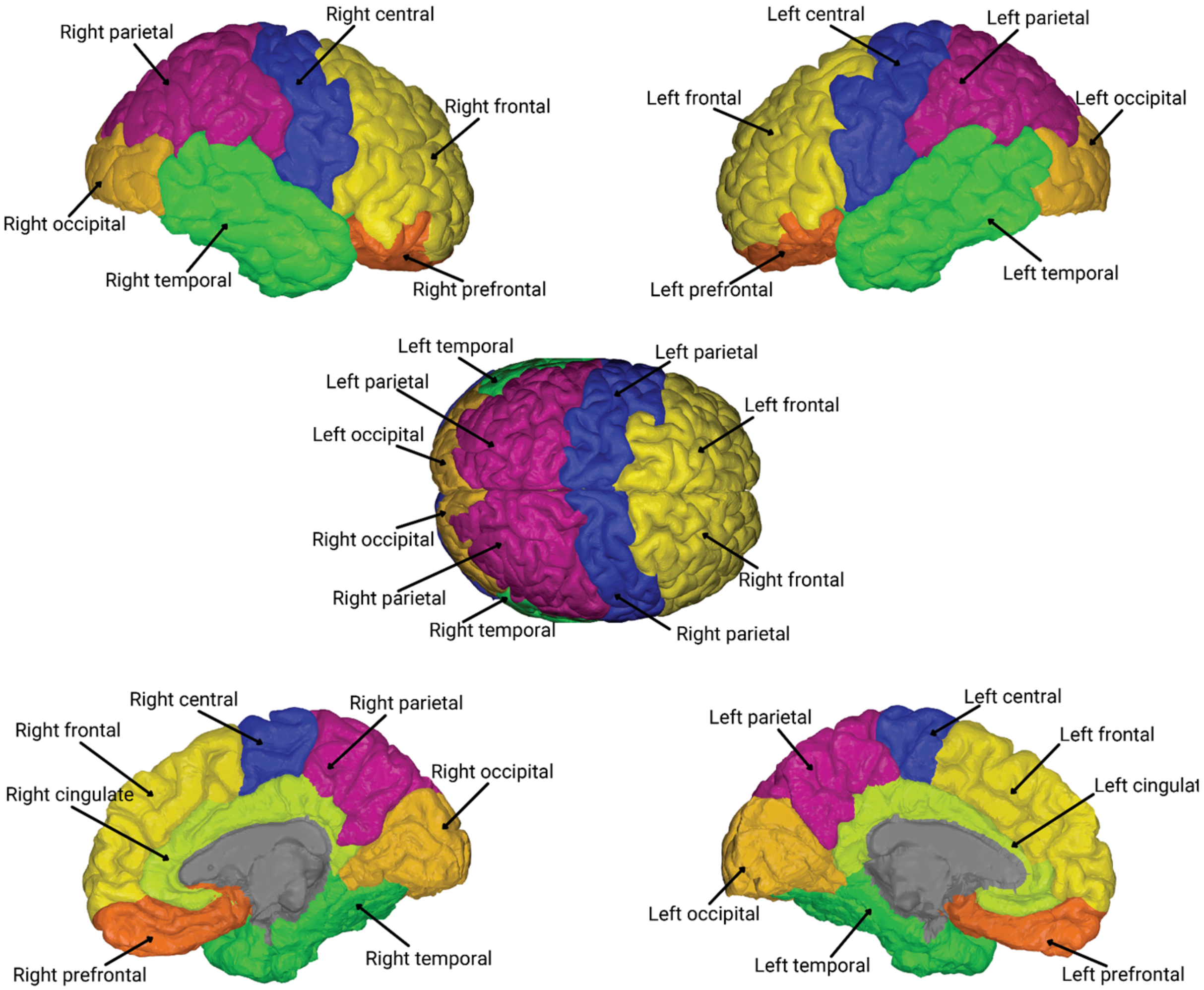

Supplementary Figure 2: The Desikan-Killiany atlas was used to group vertex parcellations following source localization. A total of 14 hierarchical regions (shown below) encompass 68 cortical nodes as described in Supplemental Table 1.

bioRxiv preprint doi: https://doi.org/10.1101/2024.06.13.598957; this version posted June 14, 2024. The copyright holder for this preprint (which was not certified by peer review) is the author/funder, who has granted bioRxiv a license to display the preprint in perpetuity. It is made available under aCC-BY 4.0 International license.

Supplementary Figure 3: Frontotemporal atlas nodes for transfer entropy estimation

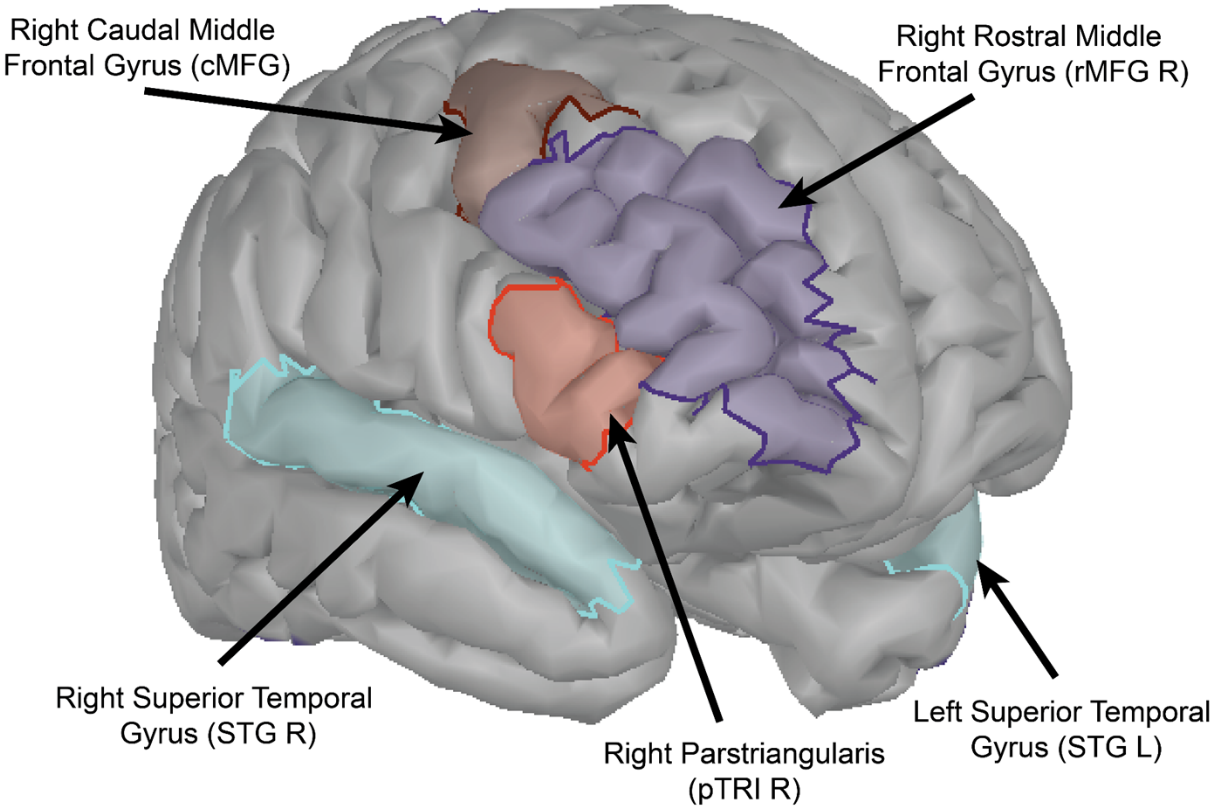

Supplementary Figure 3: Transfer entropy was calculated between frontotemporal nodes associated with significant group differences in onset and gamma ITPC. Onset ITPC was significantly increased within the caudal middle frontal R (cMFG; t=2.73, p=.001), pars triangularis R (pTRI, t =2.69, p=.011), and rostral middle frontal R (rMFG; t=2.44, p=.019) in FXS. Renyi transfer entropy was estimated between these frontal nodes and right and left superior temporal gyrus (STG), which is the node-level region that includes the auditory cortex.
